# Fiber Fabric-Reinforced Laminated Veneer Lumber (LVL) as Insulation Material for Green Buildings

**DOI:** 10.3390/ma19050872

**Published:** 2026-02-26

**Authors:** Musa Kaya, Ivan Ružiak, Ramazan Bülbül, Vedat Çavuş

**Affiliations:** 1 Yakutiye Vocational Education Center, 25100 Erzurum, Türkiye; 2 Faculty of Wood Science and Engineering, Technical University of Zvolen, T. G. Masaryka 24, 96001 Zvolen, Slovakia; ruziak@is.tuzvo.sk; 3 Department of Wood Products Industrial Engineering, Faculty of Technology, Gazi University, 06500 Ankara, Türkiye; ramazanbulbul@gazi.edu.tr; 4 Department of Forest Products Engineering, Faculty of Forestry, Kâtip Çelebi University, 35620 Izmir, Türkiye; vedat.cavus@ikcu.edu.tr

**Keywords:** laminated veneer lumber, thermal conductivity, thermal transmittance, sound absorption, sound transmission loss

## Abstract

**Highlights:**

**What are the main findings?**

- Incorporation of less than 2.5 wt.% of glass fiber fabric into poplar veneers of 81.9 wt.% bounded with 15.8 wt.% of PU adhesive reduces thermal transmittance value of the composite by 6.9%.
- Incorporation of less than 2.5 wt.% of glass fiber fabric into poplar veneers of 81.9 wt.% bounded with 15.8 wt.% of PU adhesive reduces moisture content of the composite by 6.7%.
- Incorporation of less than 2.5 wt.% of glass fiber fabric into poplar veneers of 81.9 wt.% bounded with 15.8 wt.% of PU adhesive reduces both the sound absorption coefficient and sound transmission loss of the composite by 6.7% and 15.1%, respectively.

**What are the implications of the main findings?**

- Lowering the thermal transmittance value of glass fabric-reinforced LVL makes this material proper for usage in inner and outer structures of green buildings, reducing energy demands of buildings.
- Lowered moisture content of this composite makes this material proper for usage in wet conditions, thus in bathrooms in inner structures or as outer insulation material of green buildings.
- Improved acoustic insulation properties of the composite make this material useful for applications in inner structures of green buildings, e.g., in bathrooms.

**Abstract:**

In this study Laminated Veneer Lumber (LVL)—widely used in structural wood applications—was manufactured from seven poplar veneers bonded with polyurethane (PU) adhesive and reinforced with either one sheet of glass fiber or carbon fiber fabrics. In order to determine the effects of the fiber fabrics incorporated into the structure of the produced LVLs on their thermal and acoustic insulation performance in structural applications, the thermal conductivity coefficient (λ), thermal transmittance (U), sound absorption coefficient (α), and sound transmission loss (dB) values were determined. The experimental results indicated that the thermal conductivity coefficient of the glass fiber-reinforced LVL was lower than that of both the control group and the carbon fiber-reinforced LVL. The thermal transmittance coefficient, an important indicator of thermal insulation performance in buildings, followed a similar trend. Regarding the sound absorption coefficients, the fiber fabric-reinforced LVL samples demonstrated lower coefficients compared to the control group. For sound transmission loss, no significant differences were observed among the groups, and the sound transmission loss was found to increase with frequency. Results indicate that glass fiber-reinforced LVL composites can be used as replacement of other wood-based insulating materials in green buildings which exhibit worse sound insulation or thermal insulation and which are significantly more affected by changes in relative humidity of surrounding air.

## 1. Introduction

The rapidly increasing global population, urbanization, and the growing need for sustainable resource management are driving the construction sector toward renewable, high-performance, and environmentally friendly materials. In particular, the goals of combating climate change, enhancing energy efficiency, and reducing carbon emissions have prioritized solutions that minimize the environmental footprint of building materials [1]. In this context, wood-based engineered products have assumed a critical role in modern structural applications by overcoming the inherent heterogeneity, dimensional instability, and durability limitations of natural wood [2].

LVL is an important wood-based composite produced by bonding thin wood veneers with their grain directions aligned parallel, resulting in a material with high mechanical properties and dimensional stability. Among the species commonly used in industrial LVL production, rotary-cut poplar (*Populus × euroamericana*) stands out due to its rapid growth cycle, low cost, and ease of processing [3]. However, the relatively low density and inherent natural defects of poplar wood can limit critical mechanical properties of LVL, such as tensile and bending strength.

To overcome these limitations, a common strategy is the hybridization of wood composites with high-strength synthetic fibers. Glass and carbon fibers are two of the most prominent reinforcement materials. Glass fiber offers advantages such as low cost, high tensile strength, and chemical resistance, whereas carbon fiber is known for its exceptional specific strength and stiffness, being significantly lighter than steel [4]. Recent studies have shown that reinforcing fast-growing poplar wood with carbon fiber can yield improvements of over 50% in compressive strength, elastic modulus, and deformation characteristics [5]. Similarly, panels produced by coating poplar veneers with glass fiber fabric have demonstrated notable enhancements in acoustic performance due to their inter-layer structure [6]. These findings indicate that the strategic placement of fibers in regions of high tensile stress in hybrid LVL can enhance both mechanical and acoustic efficiency [7].

The performance of hybrid LVL is determined not only by its mechanical properties but also by its physical, thermal, and acoustic characteristics, such as density, moisture content, thermal conductivity, and sound absorption capacity. The choice of adhesives is a critical factor in this regard. Compared to conventional phenol-formaldehyde (PF) adhesives, PU adhesives offer environmental and technological advantages, including the ability to cure at lower pressing temperatures, a formaldehyde-free composition, and strong bonding capability with wood surfaces [8]. The flexibility, moisture resistance, and gap-filling characteristics of PU adhesive directly influence the composite’s moisture behavior, density distribution, and acoustic performance.

Incorporating high-performance fibers and a polymeric adhesive into a wood-based composite system alters the material’s interaction with heat and sound. For instance, combining the low thermal conductivity of natural wood with the high conductivity of carbon fiber significantly changes the thermal behavior of the hybrid composite [1]. Furthermore, parameters such as panel thickness, density, fiber orientation, and porosity have been frequently reported to directly influence the sound absorption coefficient [9,10]. Studies have demonstrated that the sound absorption coefficients of natural fiber-reinforced composite panels can reach values as high as 0.8–0.9, and that these properties are closely related to variables such as density, elastic modulus, and fiber surface treatments [11,12]. Chemical modification of poplar veneers (e.g., furfuryl alcohol treatment) has also been shown to provide notable improvements in both sound insulation performance and thermal properties [13].

Another useful approach for improving properties of wood-based composites are coatings which improve antibacterial, optical and hygroscopic properties of wood materials. Article [14] reviews the possible applications of transparent wooden materials in the interior and exterior design of buildings with enhanced properties. To improve the hygroscopic performance of wood-based materials, sol–gel coatings are successfully applied to their surfaces to reduce moisture-induced deterioration. This is particularly important because wood is a material that is highly sensitive to changes in moisture content, which affect its geometry, thermal properties, and many other characteristics. These can reduce moisture content of wood similarly to fibers in wood composites and enhance antibacterial and optical parameters of wood-based products as described in articles [15,16].

This study aims to address the knowledge gap arising from the limited number of comprehensive studies on the physical, thermal, and acoustic properties of hybrid LVL composites produced from rotary-cut poplar wood (Populus × euroamericana) veneers and reinforced with glass or carbon fibers. In this context, the study experimentally investigates the effects of glass and carbon fiber reinforcement on the density (ρ), moisture content (MC), thermal conductivity coefficient (λ), thermal transmittance (U), sound absorption coefficient (α), and sound transmission loss (dB) of hybrid LVLs. Investigation of the thermal–acoustical properties of LVLs is not as common as studies about mechanical properties. On other hand, improved thermal and sound insulation properties of materials lead to lower energy demands of buildings, which is a goal of European Union grant schemes. From the results, it follows that LVLs can be used as internal insulation materials especially for glass fiber-reinforced LVLs. Locating this filler in the tensile zones guarantees optimal mechanical properties of composites, which also supports the usage of these composites as internal insulating materials of passive buildings.

## 2. Materials and Methods

### 2.1. Materials

Rotary-cut veneers from the I-214 (*Populus × euroamericana*) hybrid poplar clone were used in this study. This species, commonly employed in furniture and structural applications, has the following reported properties: oven-dry density (ODD) of 305 kg·m^−3^, air-dry density (ADD) of 332 kg·m^−3^, bending strength of 53.89 MPa, and compressive strength parallel to the grain of 28.88 MPa [17].

The veneers were selected according to first-class quality criteria, featuring straight grain and being free of knots, decay, insect damage, and fungal attack. A PU adhesive (product code 506.0, Kleiberit) was used as the bonding agent. Key properties of the materials are summarized in Table 1.

Table 1 presents the materials used in the production of LVL and some of their properties. The first number in brackets corresponds to the thickness of 1 sheet. In addition, the oven-dry and air-dry density values of the LVLs produced using these materials are given in Table 2. Based on data from Table 1, wt.% of fillers were equal to 2.3%. PU and poplar veneers correspond to the remaining 97.7 wt.%, by ratio 15.8 wt.% of PU and 81.9 wt.% of poplar veneers. The control group was prepared with 7 sheets of poplar veneers and 6 sheets of PU adhesive without fiber fabrics addition.

### 2.2. Methods

#### 2.2.1. Manufacture of LVL Composites

**Figure 1:**
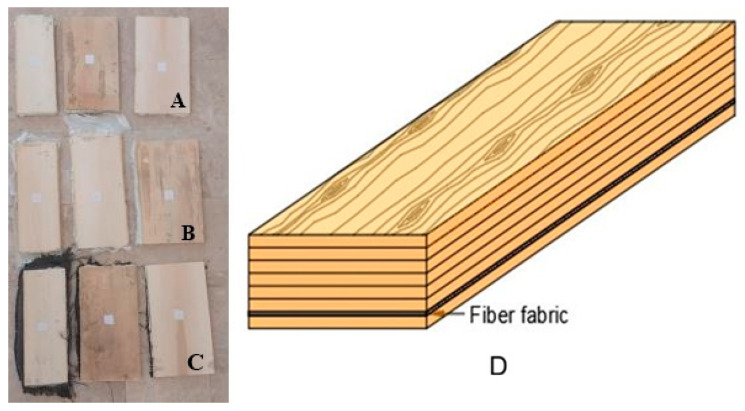
LVL samples: (**A**) control group LVL, (**B**) glass fiber-reinforced LVL, (**C**) carbon fiber-reinforced LVL. (**D**) Location of Fiber fabric in LVL composite.

Within the scope of this study, poplar veneer sheets produced by the rotary peeling method and having the dimensions given in Table 1 were conditioned to an air-dry moisture content at 20 °C and 65% relative humidity. Subsequently, in accordance with the combination shown in Figure 1, polyurethane (PU) adhesive was applied at a rate of 0.23 kg·m^−2^ to only one surface of each veneer sheet. The veneers were then pressed for 120 min under a pressure of 2.5 × 10^−4^ kg·m^−2^ using a CEMİLUSTA (Cemilusta Makina, Istanbul, Türkiye) hydraulic press. The resulting LVL panels consisted of seven poplar veneer sheets, as shown in Figure 1, and had final dimensions of 0.02 × 0.23 × 0.6 m^3^. During the manufacturing process, the fiber fabrics added to the LVL structure prior to pressing were placed between the adhesive-coated veneer sheets, specifically on the inner surface of the bottom face veneer, to produce the LVL.

#### 2.2.2. Physical Tests

**Figure 2:**
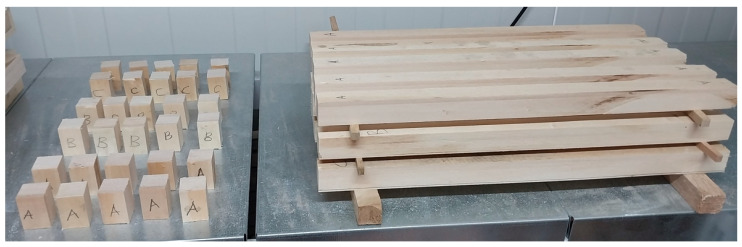
Setup for determining the ADD (**left**) and ODD (**right**) of LVL specimens. (A) Control group LVL, (B) glass fiber-reinforced LVL, (C) carbon fiber-reinforced LVL.

The physical properties of the LVLs—namely ADD, ODD, and MC—were determined. The tests followed ISO 13061-2 [18] for ρ and ASTM D4442 [19] for MC. Ten specimens (0.02 m × 0.05 m × 0.05 m) were prepared from each group and conditioned in a climate chamber at 20 ± 2 °C and 65% relative humidity (Figure 2).

ADD was determined after the specimens reached constant mass, defined as a difference of no more than 0.01 g between two consecutive weighing’s taken 6 h apart. For ODD, the specimens were dried at 103 ± 2 °C until constant mass was achieved (using the same 0.01 g criterion), with weights measured on a precision balance (0.01 g accuracy). The oven-dry weight was used to calculate both the ODD and MC.

#### 2.2.3. Technological Tests

**Figure 3:**
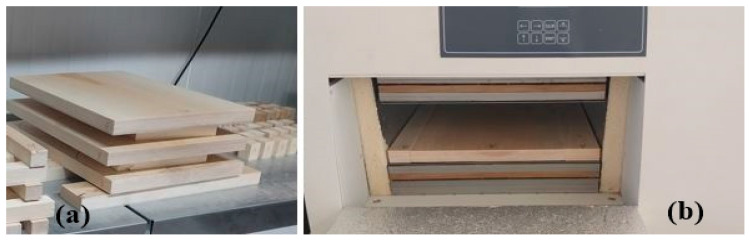
Thermal insulation measurement: (**a**) test specimens, (**b**) Linseis testing device.

Thermal Tests: The thermal insulation performance was evaluated by measuring the thermal conductivity coefficient (λ) and thermal transmittance (U-value) in accordance with TS EN 12667 [20]. Specimens (0.3 m × 0.3 m) were conditioned at 20 °C and 65% relative humidity and subsequently tested using a Linseis device (Linseis Messgeräte GmbH, Selb, Germany) (Figure 3). The Linseis device is sensitive to U-value at 0.01 W·m^−2^·K^−1^).

**Figure 4:**
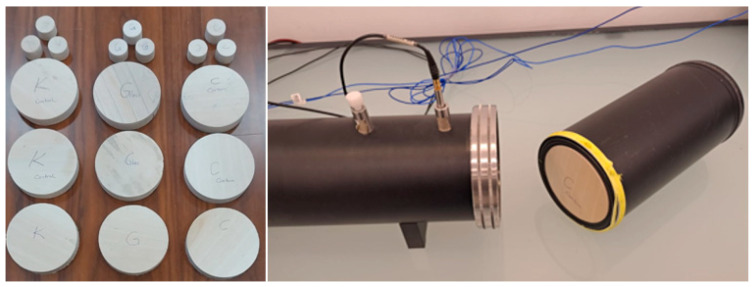
Acoustic insulation measurement of 100 mm and 30 mm diameter test specimens.

Acoustic Tests: The sound absorption coefficient (α) and sound transmission loss (dB) were determined according to ISO 10534-2 [21]. Specimens with diameters of 30 mm and 100 mm were prepared. Measurements were performed in 1/3 octave bands from 63 Hz to 6300 Hz, with primary analysis focused on the architectural acoustic range (125–4000 Hz). The tests were conducted by ŞİTEKS (Tekirdag, Türkiye) company under controlled conditions (20 °C, 65% RH, velocity of sound: 343.2 m·s^−1^, density of air: 1.2 kg·m^−3^) using an impedance tube with samples of 0.1 m and 0.03 m diameter as illustrated in Figure 4.

The frequency range of 125–4000 Hz was chosen because it corresponds to the range where human hearing is most sensitive and where most speech and common environmental noises occur. Additionally, this range is standardly used in acoustic testing of materials (e.g., ISO 10534-2, ASTM C423) and is suitable for evaluating sound absorption performance in building applications. Since our study is particularly focused on structural applications, this frequency range was adopted. In fact, these frequencies are referred to in the literature as architectural acoustics frequencies.

#### 2.2.4. Statistical Analysis

In order to determine the effects of different panel configurations on the dependent variables, a one-way analysis of variance (ANOVA) was performed. For the statistical evaluation of the data obtained in this study, SPSS Statistics 26 (IBM Corp., Armonk, NY, USA) and MSTAT-C statistical software (Michigan State University, East Lansing, MI, USA) packages were used for analysis purposes. Accordingly, a one-way ANOVA was applied to determine the effects of different fiber fabric types on the thermal and sound insulation properties of LVLs. Following the analysis of variance, Duncan’s multiple range test was employed to identify homogeneous groupings among the factors found to be statistically significant at the 5% significance level.

## 3. Results and Discussion

### 3.1. Physical Properties of LVL

The mean values for MC, ADD and ODD of the LVL materials are presented in Table 2.

Preliminary observation of Table 2 indicates variations in the physical properties among the different LVL types. To determine the statistical significance of these differences, a one-way analysis of variance (ANOVA) was performed, followed by Duncan’s multiple range test for post hoc comparison. Decrease in the MC of fiber composites is caused by a combination of PU adhesive and composite preparation. It is typical that wood composites show a lower value for MC versus basic material from which veneers are produced, mainly because of the usage of adhesives. As differences between carbon and glass fiber-reinforced LVLs are not significant, the main reason is the usage of PU adhesives.

#### Density

The ANOVA results for the air-dry and ODD values are shown in Table 3.

The ANOVA revealed a significant main effect for both the type of ρ (ADD vs. ODD) and the type of LVL (*p* < 0.05). The interaction effect (A x B) was not significant. The partial eta-squared (η^2^) value for the LVL type was 0.579, indicating that approximately 57.9% of the variance in ρ can be attributed to the incorporation of fiber fabrics. Duncan’s test was conducted to identify homogeneous groups among the significantly different LVL types, with the results presented in Table 4.

The Duncan test confirmed that the inclusion of fiber fabrics resulted in a statistically significant increase in panels ρ compared to the control group (Table 4). Although numerical differences existed between the glass and carbon fiber-reinforced LVLs, this difference was not statistically significant, as both belonged to the same homogeneous group (Group A). Furthermore, as shown in Table 2, fiber reinforcement was associated with a reduction in MC.

Poplar veneers, being low-density and thin-walled, undergo measurable micro-compression even under low pressing pressure (~2.5 × 10^−4^ kg·m^−2^). The type of reinforcement further influences this effect: carbon fiber, with its higher elastic modulus, transfers pressure more locally into the wood matrix than glass fiber, producing greater irreversible micro-compression. The curing polyurethane adhesive immobilizes this compacted structure, preventing thickness recovery during subsequent moisture loss. Consequently, LVL reinforced with carbon fiber exhibits a more pronounced density increase compared to glass fiber-reinforced counterparts, despite the reduction in moisture content.

These findings align with previous research. Several studies have reported that fiber fabric reinforcement significantly increases the LVLs ρ [22,23]. Similarly, Ref. [24] observed that adding carbon and glass fiber fabrics to various LVL layers increased ρ, and [25] reported enhanced poplar LVL ρ with carbon fiber reinforcement. The observed increase in ρ is likely due to the combined contribution of the high-density fiber fabrics, PU adhesive used for bonding, and the adhesive penetration into the fiber network.

### 3.2. Thermal and Acoustic Properties of Laminated Veneer Lumber

#### 3.2.1. Thermal Conductivity and Thermal Transmittance

The thermal conductivity (λ) and thermal transmittance (U-value) of the fiber-reinforced laminated veneer lumber (LVL) are summarized in Table 5.

In this study, it was observed that the thermal conductivity (λ) and thermal transmittance (U) coefficients of laminated veneer lumber (LVL) produced by adding different fiber fabrics to the bottom layer differed from each other. The results showed that the addition of glass fiber fabric improved the thermal insulation performance of LVL, whereas the addition of carbon fiber fabric led to increases in both the thermal transmittance (U) coefficient and the thermal conductivity (λ) coefficient. To determine whether the effects of carbon and glass fiber fabrics on the thermal insulation properties were statistically significant, the results of the one-way analysis of variance (one-way ANOVA) are presented in Table 6.

The ANOVA revealed that fiber reinforcement had a statistically significant effect on both thermal conductivity and thermal transmittance (*p* < 0.001). The partial eta-squared values (η^2^ > 0.98) indicate that approximately 99% of the variance in these thermal properties can be attributed to the type of fiber fabric used. Duncan’s multiple range test was conducted for post hoc analysis, with results presented in Table 7.

According to the results of Duncan’s test, the analysis of the thermal conductivity coefficients of LVLs reinforced with different fabrics revealed that the thermal conductivity coefficient of glass fiber-reinforced LVL decreased compared to the control group; however, this decrease was not statistically significant. In contrast, the thermal conductivity coefficient of carbon fiber-reinforced LVL was found to be statistically significantly higher than that of the control group. A similar trend was also observed in the thermal transmittance coefficients, and therefore, it can be stated that the thermal transmittance coefficients of the LVLs are consistent with their thermal conductivity coefficients.

The increase in thermal conductivity and thermal transmittance of carbon fiber-based composites lies in the higher thermal conductivity of carbon fiber versus glass fiber and versus poplar veneers and the proper weight percentage of filler. When the filler has higher thermal conductivity and the matrix and weight percentage are adequate for good adhesion of the filler to basic material, then composite thermal conductivity increases with filler wt.% until adhesion is weakened, while thermal conductivity decreases. The optimal interval of weight percentage at which filler properties affect composite properties depends on the type of filler, type of basic material, values of property of filler, and values of property of basic material. In general, when the filler’s adhesion to basic material is good, then good preparation of the composite can be realized. In the case of carbon fiber-reinforced LVL, it follows that thermal conductivity increases at this interval with wt.% of carbon fiber.

As follows from Table 7, the thermal properties of glass fiber filler LVL do not change significantly versus basic material. This is probably due to the low wt.% of filler used, as glass fiber has higher thermal conductivity versus poplar veneers at a minimum of one order higher (0.1 W·m^−1^·K^−1^ for poplar veneers and approximately 1 W·m^−1^·K^−1^ for glass fiber).

In wood and wood-based materials, thermal conductivity varies with several parameters; it generally increases with rising temperature, ρ, and MC, while the effect of thickness remains negligible [26,27]. Due to wood’s anisotropic nature, its properties, including thermal conductivity, vary considerably. Reported thermal conductivity values for softwoods and hardwoods range from 0.07 to 0.22 W·m^−1^·K^−1^ [28], while engineered wood products like OSB, LSL, and PSL typically range between 0.077 and 0.114 W·m^−1^·K^−1^ [29,30,31].

Low-density fiberboards (270–350 kg·m^−3^) with plywood coatings show thermal conductivity values of 0.062–0.087 W·m^−1^·K^−1^, while medium-density fiberboards range from 0.108 to 0.114 W·m^−1^·K^−1^ [32,33]. Carbon fiber-reinforced poplar and eucalyptus plywood panels have reported values of 0.107–0.136 W·m^−1^·K^−1^ [34]. The lower thermal conductivity of glass fiber-reinforced LVL observed in this study may be attributed to the amorphous structure of glass fiber [35]. Overall, the thermal conductivity values determined in this study align well with literature reports.

The thermal transmittance coefficient (U-value) is a crucial parameter for building thermal performance [36,37,38], with lower values indicating better insulation. Although U-value requirements vary internationally, European Union standards typically mandate wall and ceiling U-values below 0.8 W·m^−2^·K^−1^ [39]. Previous studies report U-values of 2.356 W·m^−2^·K^−1^ for 3-layer CLT panels and 1.473 W·m^−2^·K^−1^ for 5-layer CLT panels [40], while Scots pine, poplar, and poplar PSL show values of 5.59, 3.95, and 3.98 W·m^−2^·K^−1^, respectively [41,42]. The U-values obtained in this study are consistent with these literature values, with glass fiber-reinforced LVL exhibiting significantly lower U-values, likely due to the amorphous structure of glass fiber.

#### 3.2.2. Sound Absorption Coefficients

**Figure 5:**
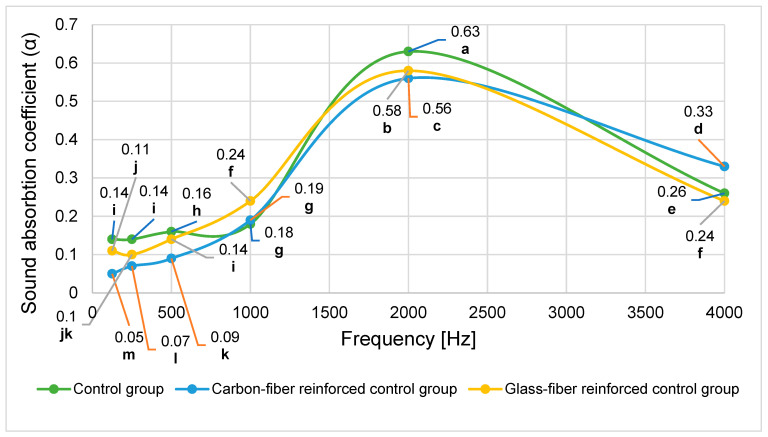
Frequency-dependent sound absorption coefficients of fabric-reinforced LVL. (…): Homogeneous groups, LSD: 0.052.

The sound absorption coefficients (α) of the used LVLs reinforced with different fiber fabrics, measured according to ISO 10534-2 [21] across architectural acoustic frequencies ([125, 250, 500, 1000, 2000, 4000] Hz), are presented in Figure 5.

Figure 5 shows distinct sound absorption behavior among the LVL types, with notably higher absorption coefficients observed at 2000 Hz across all groups. To evaluate the statistical significance of these differences, a two-way analysis of variance was performed (Table 8).

The analysis revealed statistically significant main effects for both LVL type and frequency, as well as a significant interaction between these factors (*p* < 0.001). Approximately 47.5% of the variance in sound absorption coefficients was attributable to LVL type, while frequency accounted for 99.2% of the variance. The significant interaction indicates that the effect of fiber reinforcement depends on the frequency range. Duncan’s multiple range test was conducted to identify homogeneous groups for the significant factors (Table 9 and Table 10).

The Duncan test revealed that fiber reinforcement generally decreased sound absorption compared to the control group (Table 9). The control group showed significantly higher absorption than carbon fiber-reinforced LVL, while glass fiber-reinforced LVL showed intermediate values that were not significantly different from either group. This reduction in sound absorption is likely due to the filling of wood pores with adhesive and fiber fabrics, which reduces porosity.

Sound absorption in materials occurs through viscous and thermal losses. Viscous losses result from air flow friction within material pores, while thermal losses occur when sound energy converts to heat during compression and expansion at higher frequencies [43,44]. Both mechanisms depend on the porous structure of the material. In fibrous materials, sound energy is absorbed through fiber scattering and vibration [45]. The acoustic performance of composites is influenced by characteristics such as particle size, orientation, density, and thickness [46,47]. Low-density fibrous composites typically exhibit higher sound absorption due to greater porosity, with values reaching 0.75 at 1000 Hz reported in previous studies [48]. These findings are consistent with the results observed in this study.

No statistically significant difference was found in the sound absorption coefficients (α) of LVLs reinforced with different fabrics in the low-frequency band (125–500 Hz), while the sound absorption coefficients in the medium- and high-frequency bands were found to differ statistically significantly. In conclusion, it was determined that frequency has a statistically significant effect on the sound absorption coefficient, and the highest sound absorption value was observed at 2000 Hz (Table 10). Therefore, a sound absorption coefficient of 0.590 indicates that approximately 59% of the incident sound energy at 2000 Hz is absorbed by the material, while the remaining portion is reflected or transmitted.

Sound absorption varies with frequency based on material properties, surface texture, porosity, and installation method [49]. Porous materials typically show low absorption at low frequencies, with increasing absorption at medium and high frequencies [50]. Carbon fiber composites have been reported to exhibit higher absorption in mid-frequency ranges [46], while particleboard panels show peak absorption between 1600 and 3200 Hz [51]. In this study, the LVLs demonstrated peak absorption in the 2000–2500 Hz range, which corresponds to typical human speech frequencies, indicating good acoustic performance for architectural applications.

#### 3.2.3. Sound Transmission Loss

**Figure 6:**
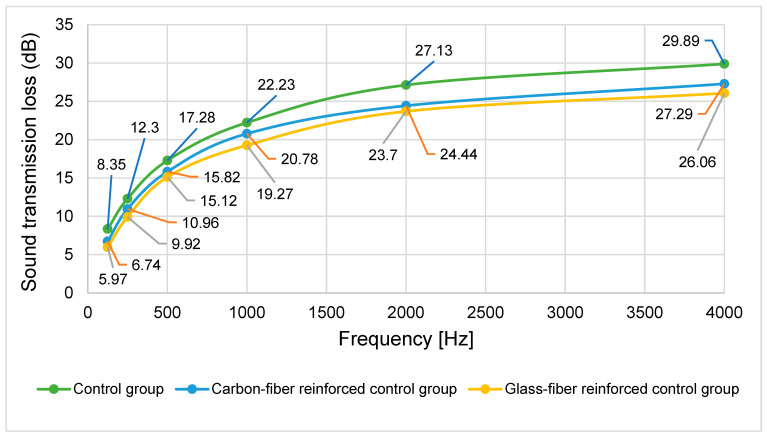
Frequency-dependent sound transmission loss of fabric-reinforced LVL.

The sound transmission loss (dB) of corresponding composite materials reinforced with various fiber fabrics, measured across architectural acoustic frequency ranges ([125, 250, 500, 1000, 2000, 4000] Hz) in accordance with ISO 10534-2 [21], is presented in Figure 6. As shown in the figure, the transmission loss varies depending on both frequency and material type. To determine whether these differences are statistically significant, a two-way analysis of variance was conducted (Table 11).

The analysis revealed statistically significant main effects for both LVL type and frequency (*p* < 0.05), accounting for 34.5% and 95.1% of the variance in sound transmission loss, respectively. However, the interaction between LVL type and frequency was not significant (*p* > 0.05), indicating that the effect of fiber reinforcement was consistent across all frequency ranges. Duncan’s multiple range test was conducted to identify homogeneous groups for the significant factors (Table 12 and Table 13).

The Duncan test revealed that fiber reinforcement significantly reduced sound transmission loss compared to the control group (Table 12). While both fiber-reinforced types showed lower transmission loss than the control, there was no significant difference between glass and carbon fiber reinforcement. This reduction may be related to changes in the dynamic behavior of the panels. Although fiber reinforcement increases stiffness, it may alter the mass law behavior and resonance characteristics, potentially reducing transmission loss in certain frequency ranges despite the increased value of ρ.

Sound transmission loss in wood materials depends on structure, density ρ, MC, thickness, surface condition, and frequency [52]. In multilayer composites, increased rigidity typically enhances sound transmission loss [53], while panel thickness, porosity, and surface layer characteristics also influence acoustic performance [54]. As limited literature exists specifically on LVL sound transmission, comparisons were made with similar composite materials, showing general consistency with previous findings.

Frequency had a substantial effect on sound transmission loss, with values increasing progressively across the measured range and peaking at 4000 Hz (Table 13). This trend follows the mass law principle, where higher frequencies result in greater transmission loss due to reduced panel vibration.

Previous studies have reported similar frequency-dependent behavior in CLT panels [55], as well as in honeycomb–core composites with carbon cladding and composite panels produced from various natural fibers such as flax and Kevlar [56,57]. In contrast, [58] different trends are observed in Canadian poplar wood, highlighting the material-specific nature of acoustic performance. It can be stated that the data obtained in this study regarding sound transmission loss are in agreement with previous studies

## 4. Conclusions

This study investigated the physical, thermal, and acoustic properties of LVL reinforced with glass and carbon fiber fabrics. The composites were produced using a cold-pressing method with fast-growing poplar veneers and PU adhesive, with fibers strategically positioned on the inner surface of the bottom layer.

The incorporation of fiber fabrics significantly influenced the physical properties of LVLs. MC decreased by 7% with glass fiber and 9% with carbon fiber reinforcement. ADD increased by 17% and 16%, while ODD increased by 18% and 15.5% in glass and carbon fiber-reinforced LVL, respectively, compared to the control group. Increase in ADD and ODD values together with a decrease in MC make these materials suitable for sound-insulating applications in inner environments with changes in relative humidity of air such as bathrooms in the form of walls, furniture and so on.

The thermal insulation performance varied considerably between fiber types. Glass fiber reinforcement had no significant effect on thermal conductivity (λ) but reduced thermal transmittance (U-value) by 6.9%. In contrast, carbon fiber reinforcement increased thermal conductivity by 11.9% and thermal transmittance by 16%. These results also show LVLs potential to be used in the internal design of green buildings as well, as in external surface materials of green buildings, thanks to a decrease in U-value.

When the sound-insulation properties of laminated veneer lumber were examined, it was determined that glass fiber fabric reinforcement caused a reduction of 6.8% in the sound absorption coefficient (α) and 15% in sound transmission loss (STL), whereas carbon fiber fabric reinforcement resulted in a decrease of 14.7% in α and 9.4% in STL. Nevertheless, an overall evaluation of the acoustic insulation performance of the LVL specimens indicated that, in all three groups, the insulation performance increased with increasing frequency levels. In this regard, the improved acoustic properties of LVLs make the use of these materials in the interior structural elements of green buildings more advantageous

In summary, while single-layer fiber fabric reinforcement primarily enhanced physical properties, the most notable technological improvement was achieved with glass fiber in thermal insulation. For productions following this design concept, incorporating glass fiber represents a cost–benefit efficient approach for enhancing thermal performance.

Future research should focus on detailed investigation of the mechanical properties of these fiber-reinforced LVL combinations and their performance in various application scenarios. Another potential field of next research is in study of wt.% of glass fibers effect on studied thermal and acoustic properties studied (by adding more layers) together with mechanical properties. Other possible future research directions cover surface coatings of LVL using sol-gels to improve optical, moisture-related properties of composites which can lead to more effective usage of LVL as self-cleaning, antibacterial and less-hygroscopic indoor products.

In future works it will be interesting to study how thw results of thermal and acoustical properties change with changes in the wt.% of parts, their number, and their position, as all these physical properties will change with these parameters. Authors in the future should perform fire-safety parameters measurements, as they are another very crucial condition for such materials to be used in buildings.

## Figures and Tables

**Table 1:** Properties of the materials used in LVL production.

Material (Quantity)	Dimensions (in cm)	ρ (kg·m^−3^)	MC (%)
Poplar Veneer (7 sheets)	[0.3; 23; 60]	338	11.3
PU (6 sheets)	[0.02; 23; 60]	1140	-
Glass Fiber (1 sheet)	[0.010; 23; 60]	2000	-
Carbon Fiber (1 sheet)	[0.011; 23; 60]	1900	-

**Table 2:** Physical properties of fiber fabric-reinforced LVLs.

Type of LVL	N	MC (%)	ADD (kg·m^−3^)	ODD (kg·m^−3^)
Control group	10	6.53 ^A^	419 ^B^	401 ^B^
Glass fiber-reinforced LVL	10	6.09 ^B^	491 ^A^	472 ^A^
Carbon fiber-reinforced LVL	10	5.94 ^B^	486 ^A^	463 ^A^

Note: Different superscript letters within a column indicate statistically significant differences (*p* < 0.05); (^A, B^): Homogeneous Group.

**Table 3:** Analysis of variance for the ADD and ODD of used LVL.

Source	Sum of Squares	df	Mean Square	F	Sig. (*p* < 0.05)	Partial Eta Squared
Type of ρ (A)	0.006	1	0.006	7.121	0.010	0.117
Type of LVL (B)	0.062	2	0.031	37.077	0.000	0.579
A × B	7.68 × 10^−5^	2	3.84 × 10^−5^	0.046	0.955	0.002
Error	0.045	54	0.001			
Total	12.548	60				
Corrected Total	0.113	59				

**Table 4:** Homogeneous groups for the ρ values of LVLs.

Type of LVL	N	ρ (kg·m^−3^)	HG
Control group	10	410	B
Glass fiber-reinforced LVL	10	481	A
Carbon fiber-reinforced LVL	10	475	A

LSD (Least significant difference): 28 kg·m^−3^, N: sample size, HG: homogeneous group.

**Table 5:** Thermal properties of fiber-reinforced LVLs.

Parameter	Type of LVL	N	Mean	COV (%)
(λ) (W·m^−1^·K^−1^)	Control group	3	0.074	1.35
	Glass fiber-reinforced	3	0.074	1.44
	Carbon fiber-reinforced	3	0.088	0.77
(U) (W·m^−2^·K^−1^)	Control group	3	3.452	2.42
	Glass fiber-reinforced	3	3.214	1.81
	Carbon fiber-reinforced	3	4.007	0.80

Mean: average value, COV: coefficient of variation.

**Table 6:** Analysis of variance for the thermal properties of fiber-reinforced LVLs.

	Source	Sum of Squares	df	Mean Square	F	Sig. (*p* < 0.05)	Partial Eta Squared
(λ) (W·m^−1^·K^−1^)	Type of LVL	0.000	2	0.000	351.50	0.000	0.992
	Error	3.41 × 10^−6^	6	5.69 × 10^−7^			
	Total	0.056	9				
	Corrected Total	0.000	8				
(U) (W·m^−2^·K^−1^)	Type of LVL	994,416.88	2	497,208	239.4	0.000	0.988
	Error	12,461.33	6	2077			
	Total	114,905,577.00	9				
	Corrected Total	1,006,878.22	8				

**Table 7:** Homogeneous groups for thermal properties of fiber-reinforced LVLs (Duncan’s test).

Types of LVL	N	(λ) (W·m^−1^·K^−1^)	HG	Types of LVL	N	(U) (W·m^−2^·K^−1^)	HG
Control group	3	0. 0741	B	Control group	3	3.45	B
Glass fiber-reinforced	3	0.0736	B	Glass fiber-reinforced	3	3.21	C
Carbon fiber-reinforced	3	0.0879	A	Carbon fiber-reinforced	3	4.01	A

LSD: 0.0014 (W·m^−1^·K^−1^), LSD: 0.09 (W·m^−2^·K^−1^).

**Table 8:** Analysis of variance for sound absorption coefficients of LVL.

Source	Sum of Squares	df	Mean Square	F	Sig. (*p* < 0.05)	Partial Eta Squared
Type of LVL (A)	0.012	2	0.006	16.299	0.000	0.475
Frequency (B)	1.578	5	0.316	847.925	0.000	0.992
A × B	0.043	10	0.004	11.570	0.000	0.763
Error	0.013	36	0.000			
Total	4.601	54				
Corrected Total	1.647	53				

**Table 9:** Homogeneous groups for sound absorption coefficients α by LVL type.

Type of LVL	N	α	HG
Control group	18	0.252	A
Glass fiber-reinforced LVL	18	0.235	AB
Carbon fiber-reinforced LVL	18	0.215	B

LSD: 0.021.

**Table 10:** Homogeneous groups for sound absorption coefficients (SAC) by frequency.

Frequency (Hz)	N	α	HG
125	9	0.100	D
250	9	0.103	D
500	9	0.130	D
1000	9	0.203	C
2000	9	0.590	A
4000	9	0.277	B

LSD: 0.030, α: sound absorption coefficient.

**Table 11:** Analysis of variance for sound transmission loss of LVL.

Source	Sum of Squares	df	Mean Square	F	Sig. (*p* < 0.05)	Partial Eta Squared
Type of LVL (A)	79.6	2	39.8	9.484	0.000	0.345
Frequency (B)	2914	5	582.8	138.850	0.000	0.951
A × B	5.31	10	0.531	0.126	0.999	0.034
Error	151.11	36	4.198			
Total	20,524	54				
Corrected Total	3150	53				

**Table 12:** Homogeneous groups for sound transmission loss STL by LVL type.

Type of LVL	N	STL (dB)	HG
Control group	18	19.53	A
Glass fiber-reinforced LVL	18	16.59	B
Carbon fiber-reinforced LVL	18	17.70	B

**Table 13:** Homogeneous groups for STL by frequency.

Frequency (Hz)	N	STL (dB)	HG
125	9	7.02	F
250	9	11.06	E
500	9	16.07	D
1000	9	20.76	C
2000	9	24.91	B
4000	9	27.78	A

LSD: 1.94 dB; STL: sound transmission loss. (dB: decibel).

## Data Availability

Data are contained within the article.

## References

[1] Asdrubali F., D’Alessandro F., Schiavoni S. (2015). A review of unconventional sustainable building insulation materials. Sustain. Mater. Technol..

[2] Bodig J., Jayne B.A. (1993). Mechanics of Wood and Wood Composites.

[3] Bal B.C. (2016). Some technological properties of laminated veneer lumber produced with fast-growing poplar and eucalyptus. Maderas. Cienc. Y Tecnol..

[4] Mallick P.K. (2007). Fiber-Reinforced Composites: Materials, Manufacturing, and Design.

[5] Gao Y., Guo L., Zhang X., Wang Y., Yang X., Fu C., Liu Z. (2022). Mechanical Properties of Fast-Growing Poplar Reinforced with Carbon Fiber. Adv. Civ. Eng..

[6] Zhang J., Liu S., Li J., Wang J., Bai H., Wei P., Liu T. (2025). Exploring the Impact of Inter-Layer Structure on Glass Fiber-Poplar Composite Board: Mechanical and Thermal Properties Analysis. Materials.

[7] Fiorelli J., Dias A.A. (2011). Analysis of the strength and stiffness of timber beams reinforced with carbon fiber and glass fiber. Mater. Res..

[8] Pizzi A. (2016). Wood products and green chemistry. Ann. For. Sci..

[9] Korjakins A., Sahmenko G., Lapkovskis V. (2025). A Short Review of Recent Innovations in Acoustic Materials and Panel Design: Emphasizing Wood Composites for Enhanced Performance and Sustainability. Appl. Sci..

[10] Mohammadi M., Taban E., Tan W.H., Din N.B.C., Putra A., Berardi U. (2024). Recent progress in natural fiber reinforced composite as sound absorber material. J. Build. Eng..

[11] Berardi U., Iannace G. (2015). Acoustic characterization of natural fibers for sound absorption applications. Build. Environ..

[12] Kudva A., Gt M., Pai K.D. (2022). Physical, thermal, mechanical, sound absorption and vibration damping characteristics of natural fiber reinforced composites and hybrid fiber reinforced composites: A review. Cogent Eng..

[13] Wu S., Xu W. (2022). Sound Insulation performance of furfuryl alcohol-modified poplar veneer used in functional plywood. Materials.

[14] Yang H., Wang H., Cai T., Ge-Zhang S., Mu H. (2023). Light and wood: A review of optically transparent wood for architectural applications. Ind. Crops Prod..

[15] Cong R., Cai T., Ge-Zhang S., Yang H., Zhang C. (2024). Fabrication of PVA–Silica SolWood Composites via Delignification and Freezing Pretreatment. Polymers.

[16] Wang Y., Ge-Zhang S., Mu P., Wang X., Li S., Qiao L., Mu H. (2023). Advances in Sol-Gel-Based Superhydrophobic Coatings for Wood: A Review. Int. J. Mol. Sci..

[17] Tunçtaner K., As N., Özden Ö. (2004). Investigation into Growth Performances, Some Technological Wood Properties and Suitability to Paper Production of Some Poplar Clones.

[18] (2021). Determination of Density for Physical and Mechanical Tests.

[19] (2025). Standard Test Methods for Direct Moisture Content Measurement of Wood and Wood-Based Materials.

[20] (2003). Thermal Performance of Building Materials and Products—Determination of Thermal Resistance by Means of Guarded Hot Plate and Heat Flow Meter Methods—Products of High and Medium Thermal Resistance.

[21] (1998). Acoustics—Determination of Sound Absorption Coefficient and Impedance in Impedance Tubes—Part 2: Transferfunction Method.

[22] Çiğdem E., Perçin O. (2022). Karbon fiber ve cam fiber ile güçlendirilmiş ısıl işlem uygulanmış lamine kaplama kerestelerin (lvl) bazı fiziksel ve mekaniksel özellikleri. Gazi Üniversitesi Mühendislik Mimar. Fakültesi Derg..

[23] Auriga R., Gumowska A., Szymanowski K., Wronka A., Robles E., Ocipka P., Kowaluk G. (2020). Performance properties of plywood composites reinforced with carbon fibers. Compos. Struct..

[24] Zdravković V., Sokolović N.M., Lovric A., Šekularac N. (2025). Physical and bending properties of beech laminated veneer lumber reinforced with carbon fiber fabric. BioResources.

[25] Wei P., Wang B.J., Zhou D., Dai C., Wang Q., Huang S. (2013). Mechanical properties of poplar laminated veneer lumber modified by carbon fiber reinforced polymer. BioResources.

[26] Sonderegger W., Niemz P. (2009). Thermal conductivity and water vapour transmission properties of wood-based materials. Eur. J. Wood Prod..

[27] Kristak L., Ruziak I., Tudor E.M., Barbu M.C., Kain G., Reh R. (2021). Thermophysical Properties of Larch Bark Composite Panels. Polymers.

[28] Demirkır M.S. (2014). The Effect of Pressing Time and Adhesive Type on the Technological Properties of Plywood Produced from Various Wood Species. Master’s Thesis.

[29] Tripathi J., Rice R. (2017). Thermal conductivity values for laminated strand lumber and spruce for use in hybrid cross-laminated timber panels. BioResources.

[30] Božiková M., Kotoulek P., Bilčík M., Kubík Ľ., Hlaváčová Z., Hlaváč P. (2021). Thermal properties of wood and wood composites made from wood waste. Int. Agrophysics.

[31] Kaya M., Bülbül R., Çavuş V. (2025). Investigation of some physical and mechanical properties of parallel strand lumber produced from willow wood. Wood Mater. Sci. Eng..

[32] Kawasaki T., Kawai S. (2006). Thermal insulation properties of wood-based sandwich panel for use as structural insulated walls and floors. J. Wood Sci..

[33] Kaya M. (2023). Mechanical and Technological Properties of Wood-Based Composites Produced with Different Layers and Materials. Ph.D. Thesis.

[34] Liu Y., Guan M. (2019). Selected physical, mechanical, and insulation properties of carbon fiber fabric-reinforced composite plywood for carriage floors. Eur. J. Wood Wood Prod..

[35] Callister W.D., Rethwisch D.G. (2020). Materials Science and Engineering: An Introduction.

[36] Park S., Shim J., Song D. (2023). A comparative assessment of in-situ measurement methods for thermal resistance of building walls under mild climate conditions. J. Build. Eng..

[37] Muhaxheri K., Muhaxheri B.B. (2023). Impact of U-Values in Evaluation of Implemented Energy Efficiency Measures and Energy Savings in Public Buildings in Context of Kosovo Legislation. WSEAS Trans. Environ. Dev..

[38] Ficco G., Iannetta F., Ianniello E., Alfano F.R.D.A., Dell’Isola M. (2015). U-Value in Situ Measurement for Energy Diagnosis of Existing Buildings. Energy Build..

[39] Ip K.C. (1994). Critical thermal transmittance (U) value for the design of green buildings. Proceedings of the Sustainable Construction: Proceedings of the First International Conference of CIB TG 16.

[40] Bülbül R., Kucuktuvek M., Keskin H. (2025). Enhancing the Thermal and Acoustic Performance of Cross Laminated Timber Panels through Perforation Technique. DREWNO.

[41] Saçli C., Altunok M., Doğan N.N. (2021). Determining the effects of modification with natural tannin and climatic conditions on thermal transmittance in some types of wood. J. Indian Acad. Wood Sci..

[42] Kaya M., Bülbül R., Türk M. (2025). Investigation of thermal and sound insulation properties of sapwood and heartwood of willow tree. BioResources.

[43] Glé P., Horoshenkov K.V., Gourdon E., Arnaud L. On the modelling of the acoustical properties of hemp concrete. Proceedings of the Acoustics 2012 Nantes Conference.

[44] Arenas J.P., Crocker M.J. (2010). Recent trends in porous sound-absorbing materials. Sound Vib..

[45] Crocker M.J., Arenas J.P. (2007). Use of sound-absorbing materials. Handbook of Noise and Vibration Control.

[46] Yan Z., Pu Z., Haijun F., Yi Z. (2019). Experiment Study on Sound Properties of Carbon Fiber Composite Material. Proceedings of the 2018 the 6th International Conference on Mechanical Engineering, Materials Science and Civil Engineering, Xiamen, China, 21–22 December 2018.

[47] Tudor E.M., Kristak L., Barbu M.C., Gergeľ T., Němec M., Kain G., Réh R. (2021). Acoustic Properties of Larch Bark Panels. Forests.

[48] Lim Z.Y., Putra A., Nor M.J.M., Yaakob M.Y. (2018). Sound absorption performance of natural kenaf fibers. Appl. Acoust..

[49] Godshall W.D., Davis J.H. (1969). Acoustical Absorption Properties of Wood-Base Panel Materials.

[50] Guan J., Li Z., Wang D. (2025). Study on the Sound Absorption Coefficient of Porous Coconut Wood Concrete Composite Materials in Concert Halls. Int. J. Chem. Mater. Sci..

[51] Bertolini M.S., Morais C.A.G., Christoforo A.L., Bertoli S.R., Santos W.N., Rocco Lahr F.A. (2019). Acoustic absorption and thermal insulation of wood panels: Influence of porosity. BioResources.

[52] Berkel A. (1970). Wood Material Technology.

[53] Arbaoui J., Schmitt Y., Pierrot J.L., Royer F.X. (2014). Effect of core thickness and intermediate layers on mechanical properties of polypropylene honeycomb multi-layer sandwich structures. Arch. Metall. Mater..

[54] Wang D., Xie S., Feng Z., Liu X., Li Y. (2020). Investigating the Effect of Dimension Parameters on Sound Transmission Losses in Nomex Honeycomb Sandwich. Appl. Sci..

[55] Kang C.W., Jang S.S., Kang H.Y., Li C. (2019). Sound Absorption Rate and Sound Transmission Loss of CLT Wall Panels Composed of Larch Square Timber Core and Plywood Cross Band. J. Korean Wood Sci. Technol..

[56] Saffari P. (2006). Sound transmission loss of honeycomb sandwich panels. Noise Control. Eng. J..

[57] Thakare P.A., Kumar N., Ugale V.B. (2019). Sound transmission loss and flexural strength assessment of hybrid composite reinforced with natural fibers and kevlar. Mater. Today Proc..

[58] Çavuş V., Kara M. (2006). Experimental Determination of Sound Transmission Loss of Some Wood Species. Kastamonu Univ. J. For. Fac..

